# Immune Modulation Mediated by Cryptococcal Laccase Promotes Pulmonary Growth and Brain Dissemination of Virulent *Cryptococcus neoformans* in Mice

**DOI:** 10.1371/journal.pone.0047853

**Published:** 2012-10-22

**Authors:** Yafeng Qiu, Michael J. Davis, Jeremy K. Dayrit, Zachary Hadd, Daniel L. Meister, John J. Osterholzer, Peter R. Williamson, Michal A. Olszewski

**Affiliations:** 1 Research Service, Veterans Administration Ann Arbor Health System, Ann Arbor, Michigan, United States of America; 2 Division of Pulmonary and Critical Care Medicine, Department of Internal Medicine, University of Michigan Medical School, Ann Arbor, Michigan, United States of America; 3 Laboratory of Clinical Infectious Diseases, National Institute of Allergy and Infectious Diseases, NIH, Bethesda, Maryland, United States of America; 4 Section of Infectious Diseases, Immunology and International Medicine, University of Illinois College of Medicine, Chicago, Illinois, United States of America; University of Sydney, Australia

## Abstract

*C. neoformans* is a leading cause of fatal mycosis linked to CNS dissemination. Laccase, encoded by the *LAC1* gene, is an important virulence factor implicated in brain dissemination yet little is known about the mechanism(s) accounting for this observation. Here, we investigated whether the presence or absence of laccase altered the local immune response in the lungs by comparing infections with the highly virulent strain, H99 (which expresses laccase) and mutant strain of H99 deficient in laccase (*lac1Δ*) in a mouse model of pulmonary infection. We found that *LAC1* gene deletion decreased the pulmonary fungal burden and abolished CNS dissemination at weeks 2 and 3. Furthermore, *LAC1* deletion lead to: 1) diminished pulmonary eosinophilia; 2) increased accumulation of CD4+ and CD8+ T cells; 3) increased Th1 and Th17 cytokines yet decreased Th2 cytokines; and 4) lung macrophage shifting of the lung macrophage phenotype from M2- towards M1-type activation. Next, we used adoptively transferred CD4+ T cells isolated from pulmonary lymph nodes of mice infected with either *lac1Δ* or H99 to evaluate the role of laccase-induced immunomodulation on CNS dissemination. We found that in comparison to PBS treated mice, adoptively transferred CD4+ T cells isolated from *lac1Δ*-infected mice decreased CNS dissemination, while those isolated from H99-infected mice increased CNS dissemination. Collectively, our findings reveal that immune modulation away from Th1/Th17 responses and towards Th2 responses represents a novel mechanism through which laccase can contribute to cryptococcal virulence. Furthermore, our data support the hypothesis that laccase-induced changes in polarization of CD4+ T cells contribute to CNS dissemination.

## Introduction

*Cryptococcus neoformans* (*C. neoformans*) is a leading cause of fatal mycosis worldwide in individuals with impaired T cell function [1], [2]. In immune compromised patients, an initial pulmonary infection of *C. neoformans* frequently results in a lethal dissemination into the central nervous system (CNS) [3]. To combat CNS dissemination, cell-mediated immune responses, especially Th1 polarization and robust expression of IFN-γ, are required [4], [5], [6], [7], [8], [9], [10]. Studies have demonstrated that Th2 adaptive immune responses are non-protective [5], [11], [12], [13]. The role of Th17 is less known, but most studies demonstrate that it can positively contribute to protection of the *C. neoformans*-infected host [14], [15], [16]. Interleukin 17A (IL-17A), a key cytokine produced by Th17 cell lineage, may itself contribute to anti-cryptococcal lung defenses [17]. In contrast, the Th2 cytokines, IL-4 and IL-13, promote cryptococcal lung infection [15], [16], [18], [19]. We have shown that the highly virulent strain of *C. neoformans*, H99, prevents induction of IFN-γ and IL-17 and enhances a non-protective Th2 in the lungs of mice [12], [16]. H99 infection in IL-4/IL-13-/- mice improves Th1 and Th17 and facilitates fungal clearance from the lung, but was insufficient to prevent CNS dissemination [16]. This suggests that highly virulent strains disseminate into the CNS using additional mechanisms beyond promoting non-protective Th2 responses in the lungs.

The pathogenesis of *C. neoformans* infection is enhanced by numerous virulence factors, many of which have been identified and shown to influence specific steps in disease onset and persistence [20]. Some factors enable yeast survival in various host tissue environments; others interfere with innate host defenses, while yet others alter adaptive defense mechanisms [21], [22], [23], [24]. Laccase, which is encoded by two laccase genes, *LAC1* and *LAC2*, is an important virulence factor for *C. neoformans*. However, *LAC1* is the predominant isoform responsible for virulence because *LAC2* basal transcript levels are low and deletion of *LAC2* does not affect virulence in mice [25], [26]. Laccase has been shown to directly protect *C. neoformans* from the antifungal activity of macrophages [27]; laccase enzymatic products include melanin pigments, iron oxidation products and prostaglandin E2 (PGE2), which all affect host defenses against *C. neoformans* [27], [28], [29]. Interestingly, an initial report found laccase to have no major effect during the pulmonary phase of infection, but to be required for brain dissemination [30]. However, in the latter study laccase deletion was performed in a moderately virulent, serotype D, *C. neoformans* strain. Newer studies utilizing highly virulent *C. neoformans* H99 (serotype A), which represents a genetically characterized standard in studies of cryptococcal virulence, demonstrated that H99 can inhibit T-cell Th17 differentiation *in vitro* through generation of PGE2 [31]. Inhibition of PGE2-mediated signaling in H99-infected mice resulted in an IL-17 and T-cell dependent improvement of mice survival. Since laccase is responsible for enzymatic biosynthesis of cryptococcal PGE2, and modulation of immune polarization is an important mechanism of the pulmonary virulence of H99 [16], we hypothesized that laccase could interfere with T-cell polarization within infected lungs and thereby contribute to pulmonary virulence of H99.

The objective of this study was to determine if laccase modulates pulmonary immune responses during infection with the highly virulent strain of *C. neoformans* H99, by influencing T cell polarization. We report that: 1) laccase contributes to pulmonary growth of highly virulent *C. neoformans* H99 through modulation of immune polarization in the infected lungs; and 2) laccase promotes CNS dissemination of H99 in part by shifting the immune response towards Th2 responses but also through a mechanism independent from T cell polarization.

## Results

### Cryptococcal Laccase Expression is Required for the Progressive Growth of a Haighly Virulent Strain of *C. neoformans* in the Lungs and Dissemination in the CNS

**Figure 1:**
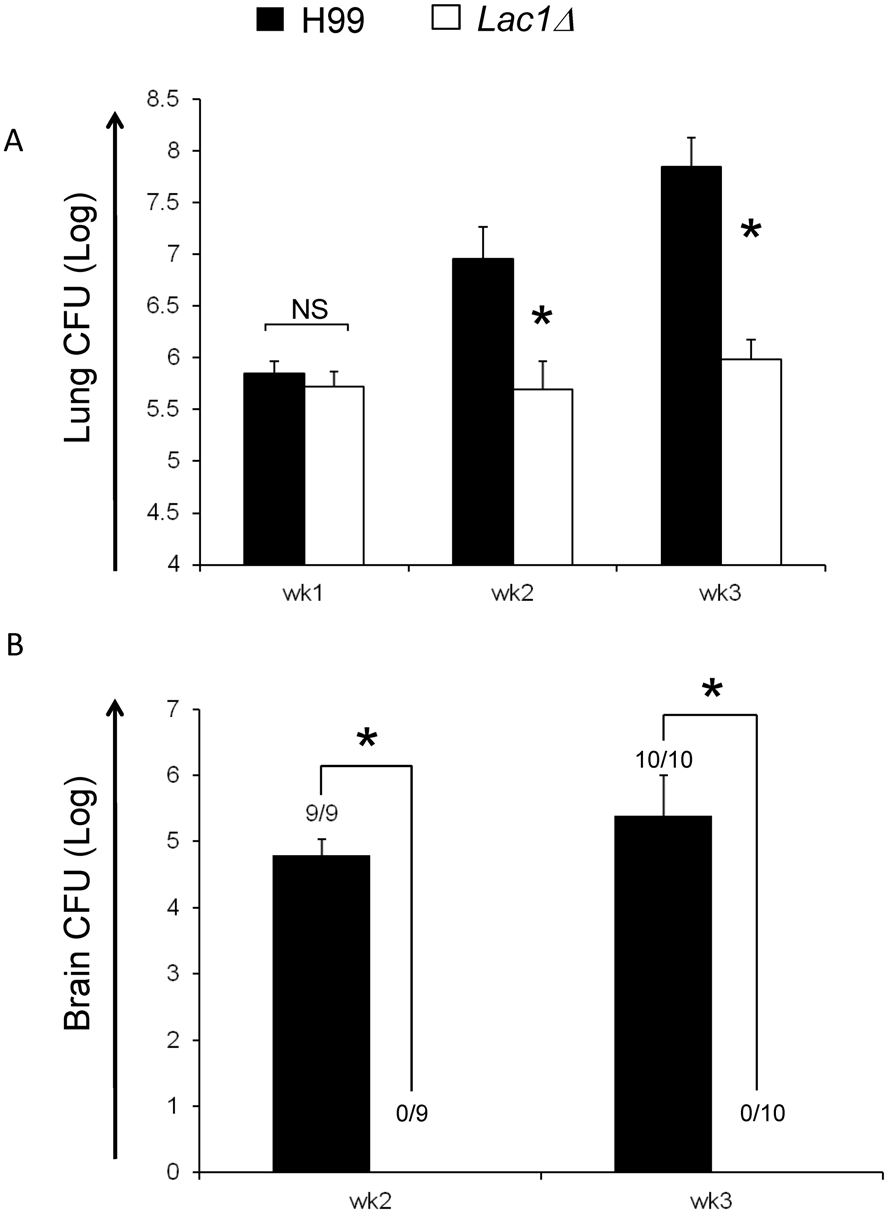
Effect of cryptococcal laccase on fungal clearance. Mice were inoculated intratracheally with 10^4^ *C. neoformans* strain H99 or *lac1Δ*. Lungs were harvested at weeks 1, 2, and 3 post-infection for analysis of fungal burden (A); brains were harvested at weeks 2, and 3 post-infection for analysis of fungal burden (B). Note the dramatically decreased fungal burden in the lungs and brains of *lac1Δ* infected mice compared with H99 infected mice. Data, mean CFU±SEM log_10_, were pooled from 2–3 separate matched experiments, *N* = 6 and above for each of the analyzed parameters; *p<0.05 in comparison between wt and mutant; NS, no significant difference between wt and mutant.

Our first objective was to compare the effect of laccase deletion from H99 on cryptococcal burden in the lungs. Mice (129/SVE) were infected intratracheally with laccase-positive (wild type strain H99, wt) and laccase-deficient (H99 with a targeted *LAC1* gene deletion, *lac1Δ*) strains. Lung CFU of mice were evaluated at weeks 1, 2, and 3 post-infection (Figure 1A). Both wt and *lac1Δ* strains showed a similar, nearly 100-fold increase above the initial inoculum of 10^4^ yeasts in the infected lungs during the first week postinfection (wpi), indicating that laccase had no effect on cryptococcal growth during the innate phase of the immune response. In contrast, while laccase-producing *C. neoformans* (H99) continued its progressive growth in the lungs during 2 and 3 wpi, the growth of *lac1Δ* strain was contained, indicating that laccase was important for progression of pulmonary infection with highly virulent *C. neoformans* during the adaptive phase of the immune response. We also evaluated the effect of laccase deletion on CNS dissemination in 129 mice. Consistent with previous data obtained with other cryptococcal strains or murine backgrounds, no CNS dissemination of the *lac1Δ* strain was observed in contrast with a massive dissemination of H99 (Figure 1B). Thus in the context of high virulence expressed by cryptococcal strain H99, laccase significantly contributes to both pulmonary virulence and *C. neoformans* dissemination into the CNS.

### Cryptococcal Laccase Expression Inhibits the Accumulation of Lymphocytes and Increases the Accumulation of Eosinophils in the Lungs

**Figure 2:**
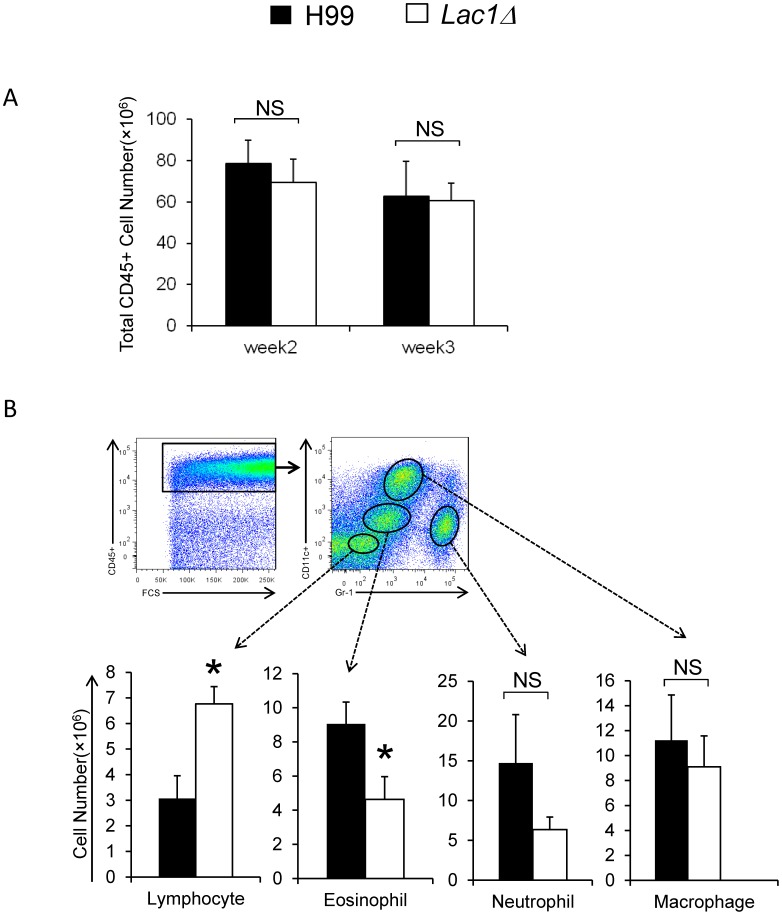
Effect of cryptococcal laccase on the recruitment of total lung leukocytes and leukocyte subsets. Lung leukocytes were isolated from mice infected with H99 or *lac1Δ*, antibody, stained and analyzed using flow cytometric analysis as per Materials and Methods. The total number of CD45+ leukocytes was determined by multiplying the frequency of CD45+ by total number cells at 2 and 3 wpi (A); Representative FACS plots show the staining strategy to isolate lung leukocyte subsets within gated populations of CD45+ leukocytess. Macrophage, Eosinophil, Neutrophil, and Lymphocyte subsets were identified based on the expression of Gr-1 vs CD11c. Total numbers of Macrophages, Eosinophils, Neutrophils, and Lymphocytse were determined by multiplying the frequency of each subset by the total number of CD45+ leukocytes at 3 wpi (B). Bars represent data (mean ± SEM) from 2–3 separate matched experiments, *N* = 6 and above for each of the analyzed parameters; *p<0.05 in comparison between wt and mutant; NS, no significant difference between wt and mutant.

To determine if changes in pulmonary growth associated with cryptococcal laccase expression were linked with changes in host responses in the lungs, lung leukocyte populations were analyzed by flow cytometry using an established gating strategy [32] and confirmed by light microscopy using differential cell counts of stained cytospun slides. No difference in pulmonary accumulation of CD45+ leukocytes were found between mice infected with wt and *lac1Δ* strains at 2 and 3 wpi (Figure 2A). Subsequently, we used differential expression of CD11c vs Gr-1 identify subsets of CD45+ leukocytes (macrophages, eosinophils, neutrophils and lymphocytes) to compare their accumulation in the infected lungs (Figure 2B). No difference in accumulation of pulmonary macrophages and neutrophils was observed between the wt and mutant strain-infected lung (Figure 2B). However, an increased accumulation of lymphocytes was observed in *lac1Δ*-infected mice compared with wt-strain infected mice (Figure 2B). Furthermore, accumulation of eosinophils, markers of aTh2 response in the lungs, was significantly reduced in *lac1Δ*-infected mice (Figure 2B).

Confirmatory experiments using light microscopy and differential cell counts redemonstrated this consistent effect of cryptococcal laccase gene deletion on pulmonary leukocyte populations. The observed frequency of eosinophils was reduced by 50 percent whereas lymphocyte numbers were doubled in the lungs of mice infected with *lac1Δ*, when compared with wt-infected mice. Collectively, our observed decrease in the accumulation of lymphocytes and increase in pulmonary eosinophils (in wt vs *lac1Δ*-infected mice) demonstrates that cryptococcal laccase expression quantitatively and qualitatively changes leukocyte recruitment during the adaptive phase of the immune response in a manner consistent with a shift towards a non-protective Th2 response.

#### Cryptococcal laccase expression promotes alternative activation of macrophages in infected lungs

**Figure 3:**
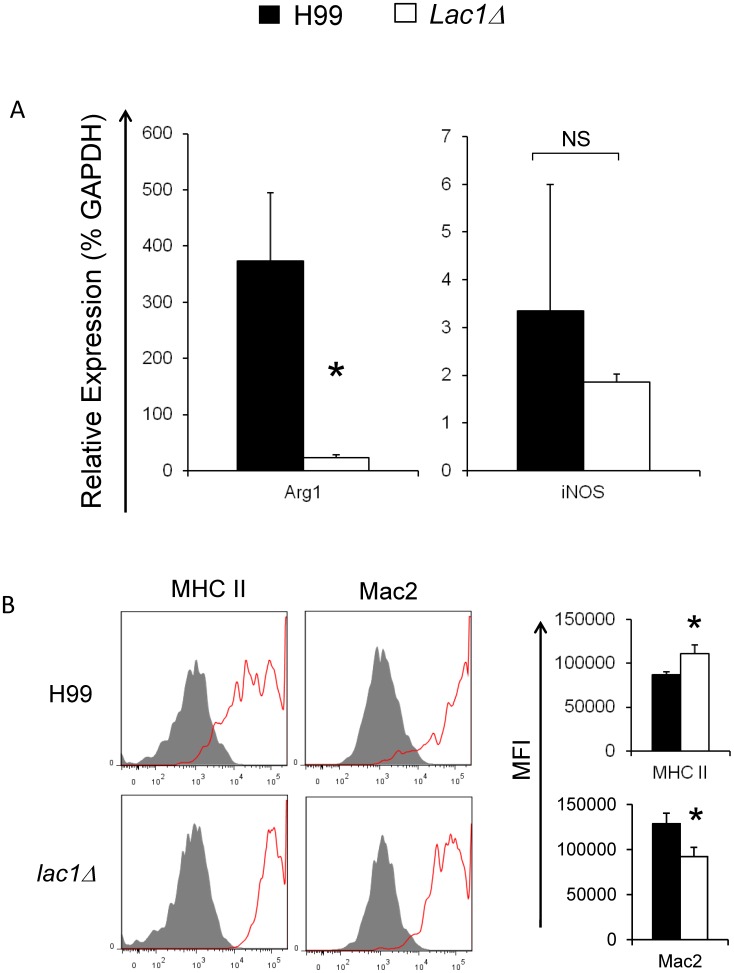
Effect of cryptococcal laccase on the activation of pulmonary macrophages. Lung leukocytes were isolated from uninfected mice or mice infected with *C. neoformans* strain H99 or *lac1Δ* at 3 wpi. (A) Pulmonary macrophages were purified as described in methods. RNA was extracted, converted to cDNA, and evaluated by qPCR to quantify alternative (Arg1) versus classical (iNOS) macrophage activation gene expression. The data were normalized to GAPDH mRNA levels and expressed as percentage of GAPDH (% GAPDH). (B) Pulmonary macrophages (CD11c+/F4/80+) were gated from CD45+ lung leukocytes by flow cytometry analysis. The activation phenotype of pulmonary macrophages was evaluated by the surface expression of MHC class II (MHC II) and Mac2. Specific antibody staining is depicted as solid lines and isotype-control staining as shaded histograms. The bar graph presents mean fluorescence intensity (MFI) of positive cells derived from these histograms. Note that H99 infection was associated with increased expression of Arg1, compared with macrophages obtained from *lac1Δ*-infected mice. In contrast, H99 infection was associated with increased expression of Mac2 and decreased expression of MHC II in the pulmonary macrophages, compared with *lac1Δ* -infected mice. Bars represent data (mean ± SEM) from 2–3 separate matched experiments, *N* = 6 and above for each of the analyzed parameters; *p<0.05 in comparison between wt and mutant; NS, no significant difference between wt and mutant.

A Th1 immune response promotes the classical activation of macrophages (M1), while a Th2 immune response promotes alternative activation of macrophages (M2) [33], [34]. This M1 vs M2 balance is critical to the containment and clearance of *C. neoformans* infections [15], [35], [36], [37]. Our next goal was to determine whether laccase expression lead to the alternative activation of pulmonary macrophages. Pulmonary macrophages obtained from wt and *lac1Δ*-infected mice were isolated at 3 wpi and examined for hallmarks of M1 (iNOS) and M2 (Arg1) activation using qRT-PCR. Macrophages from wt-infected mice showed strong up-regulation of Arg1, compared with *lac1Δ*-infected mice (Figure 3A). In contrast, no difference was found for the expression of iNOS in macrophages obtained from wt and *lac1Δ*-infected mice (Figure 3A). This outcome is consistent with a stronger M2 polarization of macrophages in the presence of laccase expression. To further explore macrophage phenotypes, we used flow cytometric analysis to analyze surface marker expression of MHC Class II (MHC II) and galectin-3 (Mac2) by pulmonary macrophages (at 3 wpi). Macrophages from wt-infected mice expressed less MHC class 2 and more galectin-3 than macrophages in the lungs of *lac1Δ*-infected mice (Figure 3B). Collectively, these data demonstrate that cryptococcal laccase expression is associated with alternative macrophage activation, providing additional evidence that cryptococcal laccase expression promotes a Th2 immune response in *C. neoformans* infected lungs.

### Cryptococcal Laccase Expression Modulates Pathology in the Lungs of Infected Mice

**Figure 4:**
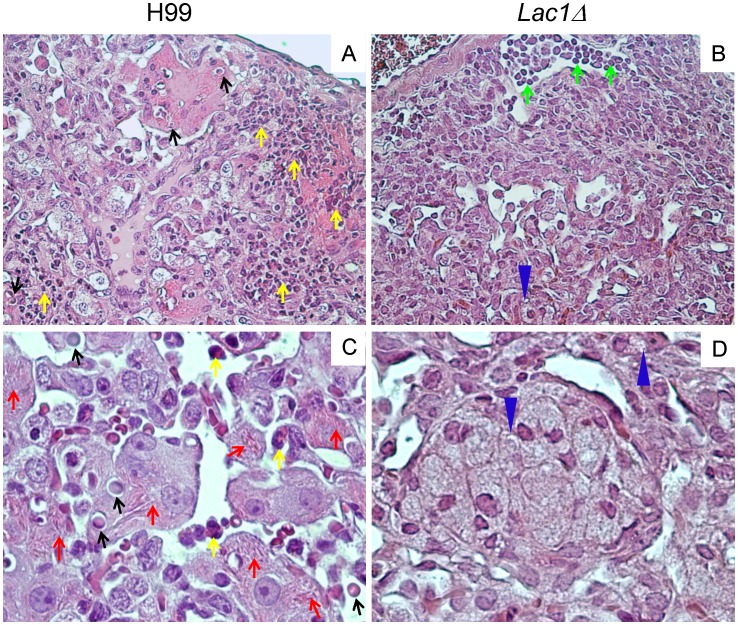
Effect of cryptococcal laccase on morphological patterns of pulmonary inflammation and pathological lesions in *C. neoformans* infected lungs. Lungs collected from H99-infected (A, C,) and *lac1Δ*-infected (B, D,) mice were perfused with buffered formalin, fixed, and processed for histology at week 3 postinfection. Representative photomicrographs of H&E + mucicarmine-stained slides taken at 40× (A, B) and 100× (C, D) objective power. Note that sections obtained from wt-infected lungs frequently revealed macrophages harboring intracellular cryptococci encircled by large capsular halos (black arrows) as compared to lung section obtained from *Lac1Δ*-infected lungs which revealed numerous macrophages with intracellular inclusions consistent with degraded cryptococcal organisms (blue arrows). Also, the extended macrophages in H99-infected mice are heavily laden with YM1/2 crystals (red arrows) which were not observed in *lac1Δ*-infected lungs. Lastly, note that H99 infection leads to the accumulation of eosinophils (yellow arrow) in the lung, compared to the accumulation of lymphocytes (green arrow) in *lac1Δ*-infected lungs.

We next performed a histopathological examination of the lungs from wt and *lac1Δ*-infected mice (at 3 wpi) to determine if cryptococcal laccase expression promoted immunopathology associated with Th2-immune responses. Consistent with our lung CFU (Figure 1A) and leukocyte subset analysis (Figure 2B), we observed a visibly higher density of cryptococcal organisms in the wt-infected mice and higher abundance of eosinophils, compared with *lac1Δ*-infected mice. Similarly, we identified few eosinophils but a relatively greater abundance of small mononuclear infiltrates in *lac1Δ*-infected lungs, consistent with improved lymphocyte recruitment (Figure 4A, B, C). Closer examination of macrophage morphology revealed that macrophages in wt-infected lungs frequently harbored intracellular cryptococci encircled by large capsular halos, consistent with live growing yeasts within alternatively activated macrophages. Furthermore, these macrophages were heavily laden with YM1/2 crystals, a protein marker of alternative macrophage activation (Figure 4C). In contrast, macrophages harboring intracellular yeast or YM 1/2 crystals were infrequently observed in *lac1Δ*-infected mice (Figure 4C, D). Rather, these macrophages often contained translucent vacuoles with smaller inclusions that likely represent the degraded microbe. Collectively, these data provide histopathologic evidence that cryptococcal laccase expression is associated with characteristic features of Th2 driven immunopathology and alternative activation of macrophages.

### Cryptococcal Laccase Expression Promotes Th2 Cytokines Expression and Impaires the Induction of Th1 and Th17 Cytokines in the Lung

**Figure 5:**
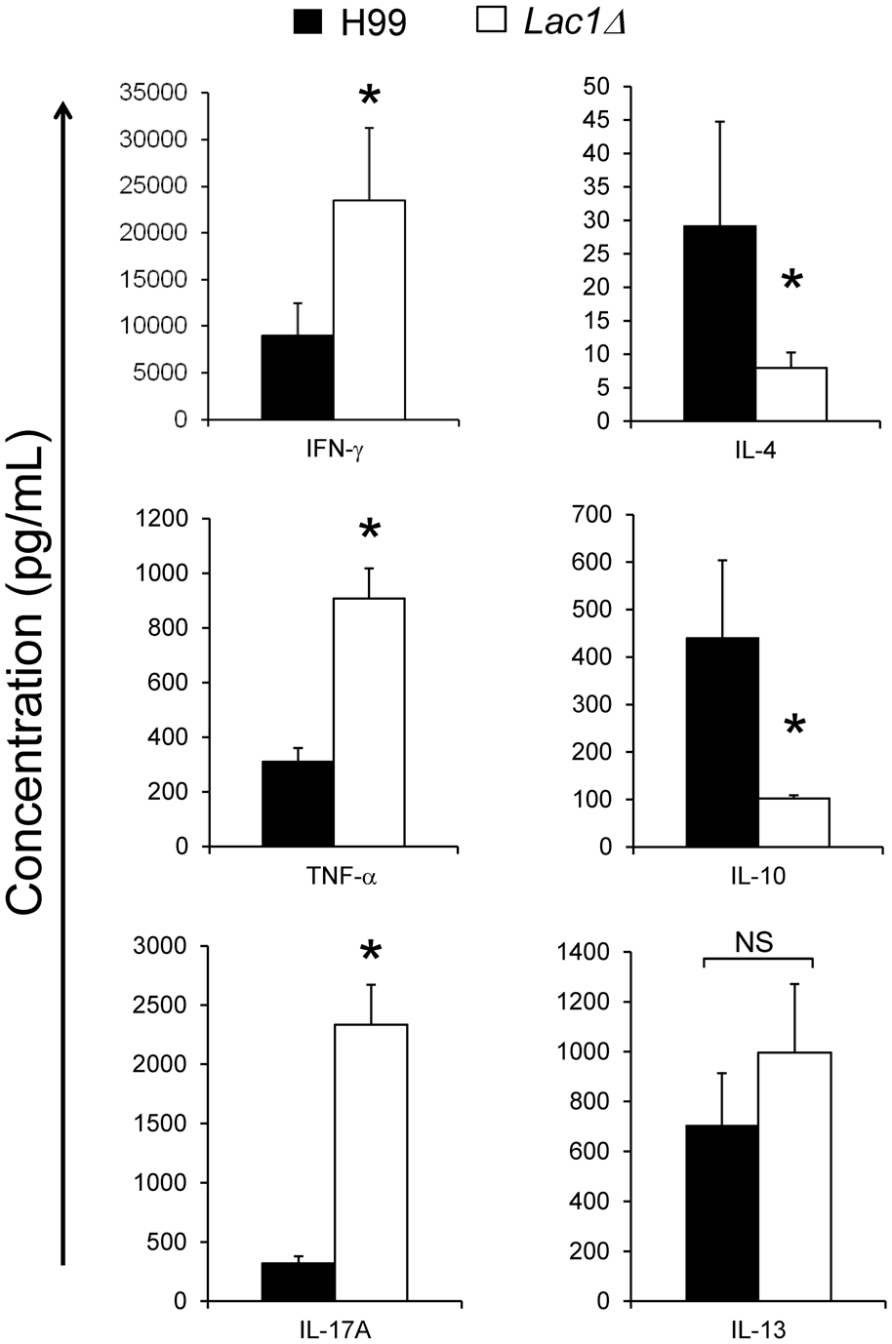
Effect of cryptococcal laccase on lung leukocyte cytokine production. Lung leukocytes were isolated from the lungs of mice infected with *C. neoformans* strain H99 or *lac1*Δ at 3 wpi and cultured for 24 h at 5×10^6^ cells/ml. Cytokine levels were evaluated by ELISA in cell culture supernatants. Bars represent mean cytokine concentration ±SEM (pg/ml). Bars represent mean ± SEM from 2 separate matched experiments, *N* = 6 and above for each of the analyzed parameters; *p<0.05 in comparison between wt and mutant; NS, no significant difference between wt and mutant.

To determine whether cryptococcal laccase affected cytokine polarization in the infected lungs, cytokine secretion by lung leukocytes was measured by ELISA at 3 wpi. We observed that cryptococcal laccase deletion significantly decreased production of Th2-type cytokines (IL-4 and IL-10) and increased production Th1-type (IFN-γ and TNF-α) and Th17 (IL-17A) cytokines compared with the production of these cytokines in wt-infected mice (Figure 5). Interestingly, no effect of laccase deletion on pulmonary IL-13 expression was observed (Figure 5).

**Figure 6:**
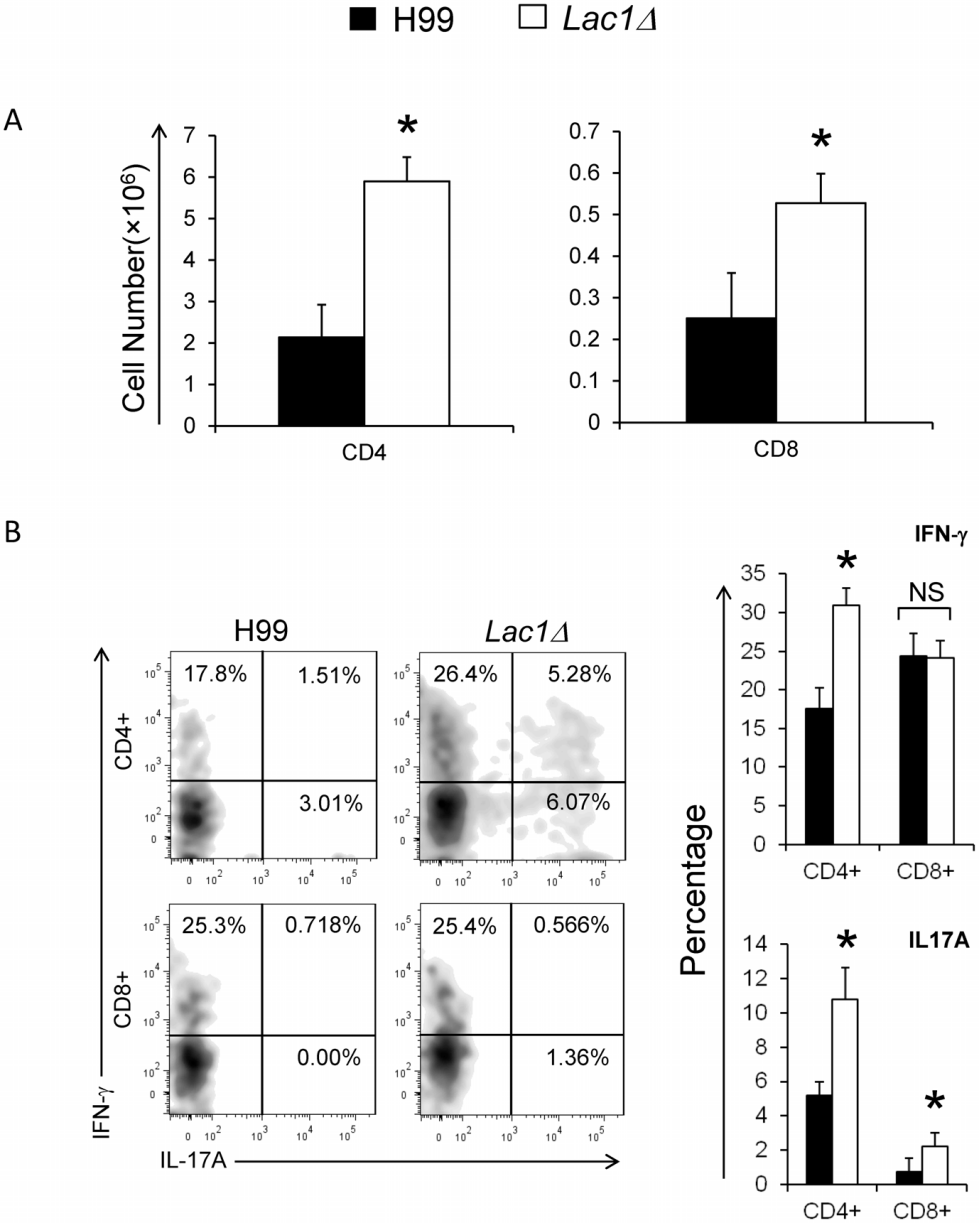
Effect of cryptococcal laccase on the recruitment and differentiation of CD4+ and CD8+ T-cells. Leukocytes were isolated from the lungs of mice infected with *C. neoformans* strain H99 or *lac1Δ* at 3 wpi. The populations of CD4+ and CD8+ T lymphocytes were identified and enumerated using flow cytometry (A); Representative plots show intracellular IFN-γ and IL-17A staining in the lung CD4+ and CD8+ T cells. The bar graphs presents the percentage of IFN-γ and IL17A positive cells within lung CD4+ and CD8+ T cells (B). Bars represent data (mean ± SEM) from 2 separate matched experiments, *N* = 6 and above for each of the analyzed parameters; *p<0.05 in comparison between wt and mutant; NS, no significant difference between wt and mutant.

To further determine the effects of cryptococcal laccase expression on T-cell responses, CD4+ and CD8+ T cells were evaluated by flow cytometric analysis at 3 wpi. We observed that cryptococcal laccase deletion significantly improved the accumulation of CD4+ and CD8+ cells in the lung of *C. neoformans* infected mice (Figure 6A), consistent with the effect on total pulmonary lymphocyte populations (Figure 2C).

Using intracellular cytokine staining, we observed that cryptococcal laccase deletion increased the frequency of IFN-γ and IL-17A-producing CD4+ T cells, compared with wt-infected mice (Figure 6B). Within the CD8+ T cell population, we also found a significant increase in IL-17A positive CD8+ T cells in *lac1Δ*-infected mice compared with that in wt-infected mice, although the percentage was lower than that in CD4+ T cells. No difference in the IFN-γ positive CD8+ T cells was observed (between wt and *lac1Δ-*infected mice). To determine whether these differences in cytokine production by T-cells were antigen specific, splenocytes from the infected mice were pulsed with cryptococcal antigens (heat killed wt yeast) and cytokine production was analyzed in cell culture supernatants. We observed a dramatic increase in antigen-induced IFN-γ production in splenocytes isolated from *lac1Δ*-infected mice compared to splenocytes from wt-infected mice (15587.5±4573.2 pg/ml vs. 5905.3±1591.4 pg/ml) and IL-17A production (1124.5±234.4 pg/ml vs. 406.3±239.2 pg/ml). Collectively, these data support our observations that cryptococcal laccase enhances Th2 responses and provides additional evidence that laccase-mediated immune modulation diminishes beneficial Th1 and Th17 cytokines.

### Cryptococcal Laccase-induced Th2 Polarization Plays a Role in Brain Dissemination

**Figure 7:**
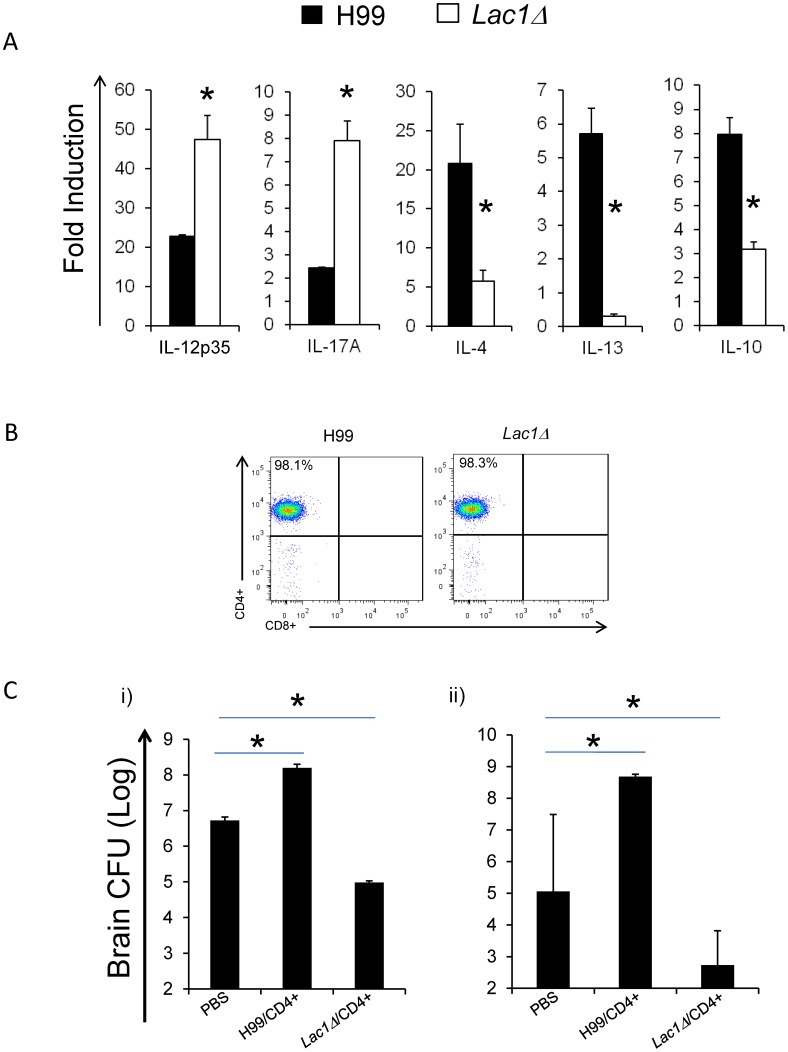
Effect of adoptively transferer CD4+ T cells on fungal clearance. Lung associated lymph nodes (LALN) were collected from uninfected (week 0) and H99 and *lac1Δ* infected mice at 2 wpi. The total cells from LALN were isolated and portion used to extract mRNA. The expression of selected “polarizing” cytokines was analyzed by qRT-PCR. Cytokine gene expression was normalized to GAPDH mRNA levels. These values were expressed as fold induction over baseline values expressed in uninfected mice (A); Remaining cells were used for magnetic bead purification of CD4+ T cells, and analyzed by flow cytometry to confirm the purity (B); Purified CD4+ T cells from H99 and *lac1Δ* infected mice at 2 wpi were adoptively transferred (i.v.) to naïve (i)) and H99 infected (at day 6 post infection) (ii)) mice. The naïve mice were challenged by 10^4^ H99 on the next day after the transfer. Mice that received PBS alone were used as controls. Brains were harvested as described in methods and results and fungal burdens evaluated (C). Bars represent data (mean ± SEM) from 2 separate matched experiments, *N* = 6 and above for each of the analyzed parameters; *p<0.05 in comparison between wt and mutant.

Our next goal was to determine if modulation of CD4+ T cell polarization was a mechanism of cryptococcal virulence associated with laccase expression. We explored this using adoptively transferred CD4+ T cells isolated from pulmonary lymph nodes from wt versus *lac1Δ*-infected mice and evaluated whether their transfer affected the course of infection with the wt strain. Since CNS infection and its progression have the highest correlation with mice survival, we focused on the effect of CD4+ cell transfer on fungal load in the brains of the infected mice. Based on the pulmonary node analysis at 1, 2, and 3 wpi the cellular responses in the pulmonary nodes occurred upstream of the changes in the lungs. We chose CD4+ T cells isolated from the LALN at 2 wpi as our source of adoptively-transferred cells. First, using comparative qPCR analysis on the total leukocyte population obtained from the LALN of wt and *lac1Δ*-infected mice, we confirmed that laccase deletion enhanced the production of cytokines associated with Th1 (IL-12) and Th17 (IL-17a) responses and decreased cytokines associated with Th2 response (IL-4, IL-13, and IL-10) (Figure 7A). Together, our data show that by 2 wpi, cryptococcal laccase strongly promoted LALN Th2 polarization and diminished LALN Th1 and Th17 immune responses.

Next, we isolated CD4+ T cells from these LALN populations. Prior to the transfers, the purity of LALN CD4+ T cells isolated from wt and *lac1Δ*-infected mice at 2 wpi was confirmed to be >98% pure (Figure 7B). Thereafter, the two groups of CD4+ T cells were transplanted into either naïve mice (Figure 7C, i)) or mice at days 6 post-infection with wt (Figure 7C, ii). The naïve mice were infected with 10^4^ wt via an intratracheal route one day after transferand fungal CFU in the brains were analyzed at 3 wpi (Figure 7C, i)). Mice receiving one of the two groups CD4+ T cells on day 6 post infection were subsequently analyzed for fungal CFU in the brains at day 16 post infection (Figure 7C, ii)). Both experimental approaches show that mice receiving CD4+ T cells from wt-infected mice showed 2–3 orders of magnitude greater fungal CFU compared with the PBS-transfer control group (Figure 7C, i) and ii)). In contrast, mice receiving CD4+ T cells from *lac1Δ*-infected mice had 2 orders of magnitude lower fungal brain burdens than the PBS-injected control group (Figure 7C, i) and ii)). Furthermore, some of the infected mice that received CD4+ T cells from wt-infected mice at 6 dpi did not survive up to day 21, but succumbed to rapidly developing menigoencephalitis. Collectively, these data indicate that transfer of Th2 polarized CD4+ cells from wt-infected mice promotes cryptococcal brain dissemination, while transfer of CD4+ T cells from *lac1Δ* infected mice inhibits cryptococcal brain dissemination. Thus, cryptococcal laccase expression interferes with protective Th1 and Th17 polarization and contributes to high CNS-tropism and meningoencephalitis-related mortality observed in mice infected with the highly virulent strain of *C. neoformans*, H99.

## Discussion

Our studies demonstrate that cryptococcal laccase can promote virulence in infected lungs and enhances CNS dissemination of a highly virulent strain of *C. neofromans*, H99. Pulmonary virulence and, in part, CNS dissemination are promoted by laccase-induced interference with T-cell polarization resulting in a shift from protective Th1 and Th17 response towards non-protective Th2 polarization. These laccase-mediated effects are evidenced by decreased lung and brain fungal burdens and profound changes in all major characteristics of the polarized pulmonary and systemic immune response. Specifically, in mice infected with the laccase-deficient strain of H99, *lac1Δ,* we observe: a) decreased eosinophil accumulation; b) increased accumulation of lymphocytes including CD4+ and CD8+ T cells; c) increased frequencies of CD4+ expressing robust IFN-γ and IL-17A; d) overall changes in major groups of Th-signature cytokines elicited by lung and thoracic lymph node leukocytes favoring Th1 and Th17 responses over Th2 response; and e) a shift in macrophage polarization profile towards the more protective M1 phenotype. Our data further demonstrate that this mechanism of immunological interference affected systemic immunity, documented by: a) changes in splenocyte responses to cryptococcal antigen; and b) an observed CNS protective effect of transferred T cells polarized in the presence of laccase negative yeast versus the detrimental CNS effect of transferred T-cells polarized in the presence of laccase-producing yeast. Taken together, modulation of immune polarization induced by cryptococcal laccase expression is an important mechanism for both pulmonary and systemic virulence expressed by highly virulent *C. neoformans*.

As previously demonstrated, cryptococcal laccase contributed to *C. neoformans* virulence; however, it was not found to affect pulmonary growth or characteristics of the cellular immune response to a moderately virulent strain in a murine model of cryptococcal pneumonia [30]. These outcomes were consistent with laccase facilitating *C. neoformans* escape from the infected lung, but not its role in other aspects of pathogenesis during cryptococcal infection [30]. However, laccase is a crucial enzyme required for biosynthesis of melanin and prostaglandin E2 by *C. neoformans* [29], [38], both of which are known to profoundly affect interaction of the microbe with the mechanisms of host defense. Thus we suspected that different biological conditions (for example the timing of laccase expression by a highly virulent strain of *C. neoformans*) may further unmask the potential of laccase to modulate pulmonary and/or systemic host responses. Unlike many less virulent strains, highly virulent strain H99 displays a strong immunomodulatory potential [12], [16]. Consistent with this notion, one recent study demonstrated that laccase expression by H99 can affect lymphocyte polarization *in vitro* and interfere with survival of infected mice due to inhibition of IL-17 production and Th17 polarization [31]. This finding contributed to our hypothesis that laccase could play a more prominent role in the lungs during an *in vivo* infection with highly virulent strains such as H99. Our present data demonstrate that laccase is required for progressive growth of strain H99 in the lungs, providing novel evidence that laccase can significantly contribute to pulmonary virulence of *C. neoformans*. We identified this laccase-dependent effect during the adaptive phase of the immune response, suggesting that cryptococcal laccase is one of the factors triggering the development of a non-protective immune deviation. The effect of laccase is characterized by evidence of robust Th2 polarization including the accumulation of lung eosinophils, enhanced production of Th2 cytokines IL-4 and IL-10 by lung leukocytes, M2 polarization of lung macrophages, and the induction of Th2 cytokines IL-4, IL-13 and IL-10 by leukocytes residing in lung associated lymph nodes.) Furthermore, laccase appears to shift the adaptive immune response away from Th1 and Th17 polarization since the absence of laccase was associated with increased pulmonary recruitment of Th1 and Th17 T cells and increased production of IFN-γ TNF-α, IL-12 and IL-17A by lung and/or pulmonary lymph node leukocytes. Since the robust Th1 and Th17 response supports pulmonary containment of H99, our data support the notion that cryptococcal laccase expression promotes pulmonary growth of the highly virulent strain H99 by causing an immunological shift from a protective Th1/Th17 polarization profile, towards a detrimental Th2 response.

Although our study identifies a novel role for cryptococcal laccase in the pathogenesis of cryptococcal infections, laccase is not the only factor that shows a potential to modulate an adaptive response in H99 strain. Such roles have been reported for cryptococcal urease and phospholipase B1 (*PLB1*) [39], [40]. At this point, it is not clear if these factors contribute to immune deviation via separate mechanisms or if these enzymes are all required for a generation of specific product(s) that, in turn, skew(s) the adaptive immunity. However, the expression of the other immunomodulatory factors by *lac1Δ* could explain why only partial protection was achieved in the lungs following laccase deletion. For example, we did not observe a progressive clearance of *lac1Δ*, although crytpococcal control in the lungs improved following laccase deletion. We also did not see a complete switch of macrophage polarization from M2 to M1, since the decrease in arginase expression was not accompanied by increase in iNOS expression. Future studies are needed to clarify how immunomodulatory cryptococcal factors interact to induce the strong pro-Th2 polarizing properties displayed by strain H99. Based on the biological substrate relationship between laccase and *PLB1* it is likely that these two factors could work together to cause immune deviation through their contribution to PGE2 synthesis as laccase-derived fungal prostaglandin (PGE2) [29], [38] is a known immunomodulatory factor, that can interfere with immune polarization during *C. neoformans* infections [31]. A novel function of fungal PGE2 reported recently is to inhibit IL-17 expression during T cell differentiation and cryptococcal infection [31]. Consistent with this finding, our data showed that laccase deletion increases the expression of IL-17A and Th17 polarization in the lungs, LALN and spleens of *C. neoformans*-infected mice.

An additional mechanism by which laccase could interfere with the adaptive response is through biosynthesis of melanin although this is less likely in lung due to low levels of cathecholamine substrates in lung [41]. Laccase is the major enzyme required for cryptococcal melanin production and high expression of cryptococcal melanin is linked to down-regulation of TNF-α and subsequent development of non-protective immune deviation [42], [43].

Although, our studies focused predominantly on T-cell related effects of laccase in host-pathogen interactions of *C. neoformans*, it becomes quite apparent that laccase-mediated virulence is linked to a group of biological effects of which one is linked to a mechanism of immune T-cell polarization. Previous studies defined a role for laccase that was related to enhanced extrapulmonary dissemination, which was not linked to the level of pulmonary growth or immune polarization. Our study with a highly virulent strain demonstrates that laccase expression was also strongly linked to CNS dissemination. However, the effect of laccase deletion on CNS dissemination was more powerful than the effect on pulmonary host defenses. While laccase deletion improved pulmonary control of *C. neoformans* (Fig. 1A) it prevented CNS dissemination completely (Fig. 1B), suggesting that apart from immunomodulatory effects, laccase could also have other distinct function that contributed to CNS dissemination. Our adoptive transfer experiment further documents that CD4+ T-cells from H99-infected LALN were Th2 polarized and promoted CNS dissemination. In contrast, CD4+ T-cells from *lac1Δ*-infected LALN were more Th1 polarized and decreased CNS dissemination. These results demonstrate that Th2 polarization mediated by cryptococcal laccase is an important factor in CNS dissemination of *C. neoformans* H99. However, the adoptive transfer of Th1/Th17 cells from the nodes of mice infected with *lac1Δ* did not abolish CNS dissemination of H99, further supporting the notion that laccase has an additional role in pathogenesis of CNS dissemination beyond its immunomodulatory effects.

At this time is not clear why laccase did not display detectable immunomodulatory effects in the previous study [30]. We suspect that this could be related to differences between cryptococcal strains and their level of laccase expression. In fact, differences in melanin production *in vitro,* which is a readout of laccase activity, were reported to contribute to the differential level of virulence expressed by different cryptococcal strains [42]. Another possibility is that the immunomodulatory effects of moderately virulent strain system could be unmasked in other strains of mice. We do not favor this hypothesis, since we could also observe effects of laccase on pulmonary growth in other strains of mice including CBA/J mice used for the moderately virulent strain studies [30]. Future studies comparing effects of laccase deletion in different cryptococcal strains and its interplay with other virulence factors are needed to clarify this point.

In summary, our findings provide insight into the role of cryptococcal laccase in a highly virulent strain of *C. neoformans*. Laccase contributes to pulmonary growth of *C. neoformans* H99 through modulation of immune polarization in the infected lungs and promotes CNS dissemination of H99, in part, by shifting the immune response towards Th2, but also via a mechanism independent of T cell polarization. Furthermore, this study provides evidence that Th2 CD4+ T-cells can contribute to CNS dissemination. Collectively, these results reveal that laccase expression can promote the pathogenesis of cryptococcal infections in the lung and within the CNS via multiple mechanisms, some of which involve modulation of T-cell responses.

## Materials and Methods

### Ethics Statement

These studies were performed in compliance with the protocols approved by the institutional R&D, animal studies and research safety committees of the VA Ann Arbor Health System. Ann Arbor VA Animal Studies Committee approved these studies (protocol number 0512-025) in strict accordance with the recommendations in the Guide for the Care and Use of Laboratory Animals of the National Institutes of Health. All manipulations involving live mice were performed under general anesthesia and endpoint criteria were used for survival experiments to humanely euthanize moribund animals. All efforts were made to ensure proper care for the animals and to minimize suffering.

### Mice

Female 129 (SVE) mice were obtained from Taconic Farms Inc (Germantown, NY). Mice were aged to 8 to 10 weeks at the time of infection.

### C. Neoformans

*C. neoformans* strain H99 (ATCC 208821) and *lac1Δ* mutant (H99 with a targeted *LAC1* gene deletion, as descriped by Dr. J. Andrew Alspaugh from Duke University, Durham, NC [25]) was recovered from 10% glycerol frozen stocks stored at -80°C and grown to stationary phase at 37°C in Sabouraud dextrose broth (1% neopeptone, 2% dextrose; Difco, Detroit, MI) on a shaker. The cultures were then washed in non-pyrogenic saline (Travenol, Deerfield, IL), counted on a hemocytometer, and diluted to 3.3×10^5^ yeast cells/ml in sterile non-pyrogenic saline.

### Intratracheal Inoculation of *C. neoformans*

Mice were anesthetized via intraperitoneal injection of Ketamine/Xylazine (Ketamine/Xylazine 100/6.8 mg/kg/BW) and were restrained on a foam plate. A small incision was made through the skin covering the trachea. The underlying salivary glands and muscles were separated. Infection was performed by intratracheal injection of 30 µl (10^4^ CFU) via 30-gauge needle actuated from a 1 ml tuberculin syringe with *C. neoformans* suspension (3.3×10^5^/ml). After inoculation, the skin was closed with cyanoacrylate adhesive, and the mice were monitored during recovery from the anesthesia.

### Organ CFU Assay

For determination of microbial burden in the lungs, small aliquots of dispersed lungs were collected following the digestion procedure. For determination of brain CFU, the brains were dissected using sterile instruments, placed in 2 ml of sterile water and homogenized. Series of 10-fold dilutions of the lung and brain samples were plated on Sabouraud dextrose agar plates in duplicates in 10 µl aliquots and incubated at room temperature. *C. neoformans* colonies were counted 2 days later and the number of CFU was calculated on a per-organ base.

### Lung Leukocyte Isolation

The lungs from each mouse were excised, washed in RPMI, minced with scissors, and digested enzymatically at 37°C for 30 minutes in 5 ml/mouse of digestion buffer [RPMI, 5% FBS, penicillin and streptomycin (Invitrogen, Grand Island, NY); 1 mg/ml Collagenase A (Roche Diagnostics, Indianapolis, IN); and 30 µg/ml DNase (Sigma, St. Louis, MO)] and processed as previously described [44], [45]. The cell suspension and tissue fragments were further dispersed by repeated aspiration through the bore of a 10 ml syringe and were centrifuged. Erythrocytes in the cell pellets were lysed by addition of 3 ml of NH_4_Cl buffer (0.829% NH_4_Cl, 0.1% KHCO_3_, and 0.0372% Na_2_EDTA, pH 7.4) for 3 minutes followed by a ten-fold excess of RPMI. Cells were resuspended and a second cycle of syringe dispersion and filtration through a sterile 100 µm nylon screen (Nitex, Kansas City, MO) was performed. The filtrate was centrifuged for 25 minutes at 1500 g in the presence of 20% Percoll (Sigma) to separate leukocytes from cell debris and epithelial cells. Leukocytes pellets were resuspended in 5 ml of complete RPMI media, and enumerated on a hemocytometer following dilution in Trypan Blue (Sigma).

### Lung-associated Lymph Node Leukocyte Isolation

Individual lung-associated lymph nodes (LALN) were excised. To collect LALN leukocytes, nodes were dispersed using a 3 ml sterile syringe plunger and flushed through a 70 µm cell strainer (BD Falcon, Bedford, MA) with complete media into a sterile tube, as described previously. After being centrifuged at 12,000 rpm/min for 10 min, the supernatant was removed and the cell pellets were saved at -70°C for gene expression analysis by real-time reverse transcription PCR.

### Pulmonary Cytokine Production

Isolated lung leukocytes were diluted to 5×10^6^ cells/ml and were cultured in 24-well plates with 2 ml of complete RPMI medium at 37°C and 5% CO_2_ for 24 h. Supernatants were separated from cells by centrifugation, collected, and frozen until tested. The cytokines TNF-α, IFN-γ, IL-17A, IL-4, IL-10, and IL-13 were quantified by ELISA using DuoSet kits (R&D Systems, Minneapolis, MN) following the manufacturer’s specifications. All plates were read on a Versamax plate reader (Molecular Devices, Sunnyvale, CA).

### Antigen-specific Cytokine Production by Splenocytes

Spleens were excised and dispersed using a 3 ml sterile syringe plunger and flushed through a 70 µm cell strainer (BD Falcon, Bedford, MA) with complete media. Isolated spleen cells were diluted to 5×10^6^ cells/ml and were cultured in media alone or with heat-killed *C. neoformans* (H99) in a ratio of 1∶2 in 24-well plates with 2 ml of complete RPMI medium at 37°C and 5% CO_2_ for 48 h. Supernatants were stored and analyzed for cytokine levels as described above. The antigen-specific cytokine production was calculated as a net gain of cytokine level compared to unstimulated controls of the same sample.

### qRT-PCR

Total RNA was prepared using RNeasy Plus Mini Kit (Qiagen, Valencia, CA, USA) and first-strand cDNA was synthesized using SuperScript™III (Invitrogen, Carlsbad, CA, USA) according to the manufacturer's instructions. Cytokine or other mRNA was quantified with SYBR Green-based detection using an MX 3000P system (Stratagene, La Jolla, CA) according to the manufacturer's protocols. Forty cycles of PCR (94°C for 15 s followed by 60°C for 30s and 72°C for 30s) were performed on a cDNA template. The data were normalized to GAPDH mRNA levels.

### Antibodies and Flow Cytometric Analysis

For flow cytometry experiments, antibodies were purchased from Biolegend, San Diego, CA, including rat anti-murine CD16/CD32 (Fc block), rat anti-murine CD45 conjugated to allophycocyanin (APC), hamster anti-murine CD11c or CD8 conjugated to pacific blue (PB), rat anti-murine CD11b or CD4 conjugated to allophycocyanin-Cy7 (APC-Cy7), rat anti-murine Gr-1 conjugated to phycoerythrin-Cy7 (PE-Cy7), rat anti-murine CD3, CD19 or IFN-γ conjugated to PerCP-Cy5.5, rat anti-murine F4/80 or CD19 conjugated to Fluorescein isothiocyanate (FITC), and rat anti-mouse MHC class II, Mac2 or IL-17A conjugated to PE.

Antibody cell staining was performed as previously described [40]. Data were collected on a FACS LSR2 flow cytometer using FACSDiva software (Becton Dickinson Immunocytometry Systems, Mountain View, CA). A minimum of 20,000 cells were evaluated from a predominantly leukocytic population identified by CD45+-stained cells per sample. The flow data is analyzed by FlowJo software (Tree Star Inc., San Carlos, CA). Total numbers of each cell population were calculated by multiplying the frequency of the population by the total number of leukocytes (the percentage of CD45+ cells multiplied by the original hemocytometer count of total cells). The frequencies of leukocyte subsets were obtained by the expression of Gr-1 vs CD11c from the gated leukocytes. The outcomes of flow cytometry readouts have been validated by microscopy of concurrent leukocyte preparations on cytospun slides and alternative antibody staining procedures.

### Intracellular Cytokine Staining

Total lung cells were stimulated for 4–6 hours at 37°C with PMA (50 ng/ml) (Sigma) and ionomycine (1 µg/ml) (Sigma) in the presence of GolgiPlug (BD Pharmingen) according to the manufacturer's protocols. After stimulation, surface molecules of cells were stained as described above. Subsequently, intracellular molecules were stained using the BD Cytofix/Cytoperm kit according to the manufacturer’s instructions (BD Pharmingen).

### Adoptive T Cells Transfer

Lung-associated lymph nodes (LALN) leukocytes were isolated as described above. Subsequently, LALN’s CD4+ T cells from H99 and *lac1Δ* infected mice were purified by negative selection of CD4+ T cells using the AutoMACS magnetic purification system (Miltenyi Biotec, Bergisch Gladbach, Germany). The purity of CD4+ T cells was assessed by Flow cytometric analysis. A total of 0.5×10^6^ purified LALN’s CD4+ T cells from H99 and *lac1Δ* infected mice were injected intravenously (i.v.) into naïve and H99 infected (at day 6 post infection) mice, and mice that received sterile PBS alone were used as control. The naïve recipients were challenged by 10^4^ H99 via intratracheal route on another day after transfer. At the end of the experiment, the brain was harvested to analyze the fungal burden as described above.

### Histology

Histology was performed as previously described [16]. Briefly, lungs were fixed by inflation with 1 ml of 10% neutral buffered formalin, excised *en bloc*, and immersed in neutral buffered formalin. After paraffin embedding, 5-µm sections were cut and stained using hematoxylin-eosin (H&E) with mucicarmine. Sections were analyzed with light microscopy and microphotographs taken using Digital Microphotography system DFX1200 with ACT-1 software (Nikon Co, Tokyo, Japan).

### Calculations and Statistics

Statistical significance was calculated using Student’s t-test for individual paired comparisons or one-way ANOVA, whenever multiple groups were compared. All values are reported as means ± standard errors (SEM). Means with *p* values of <0.05 were considered significantly different. All statistical calculations were performed using Primer of Biostatistics (McGraw-Hill, NY).
